# The role of arts in moderating mental health-related stigma: views of early career psychiatrists and trainees from different parts of the world

**DOI:** 10.3389/fpsyt.2024.1293142

**Published:** 2024-02-20

**Authors:** Sarah El Halabi, Ely Fish, Mahsa Boroon, Renato de Filippis, Samer El Hayek, Amine Larnaout, Dorottya Ori, Mariana Pinto da Costa, Rodrigo Ramalho, Ramdas Ransing, Fajar Raza, Mohammadreza Shalbafan

**Affiliations:** ^1^Westchester Medical Center Psychiatry Department, Valhalla, NY, United States; ^2^Department of Psychiatry, School of Medicine, Alborz University of Medical Sciences, Karaj, Iran; ^3^Psychiatry Unit, Department of Health Sciences, University Magna Graecia of Catanzaro, Catanzaro, Italy; ^4^Medical Department, Erada Center for Treatment and Rehabilitation in Dubai, Dubai, United Arab Emirates; ^5^Department of Psychiatry, Razi Hospital, Faculty of Medicine, University of Tunis, El Manar, Tunisia; ^6^Institute of Behavioral Sciences, Semmelweis University, Budapest, Hungary; ^7^Department of Mental Health, Heim Pal National Pediatric Institute, Budapest, Hungary; ^8^Institute of Psychiatry, Psychology and Neuroscience, King’s College London, London, United Kingdom; ^9^Department of Social and Community Health, University of Auckland, Auckland, New Zealand; ^10^Department of Psychiatry, Clinical Neurosciences and Addiction Medicine, All India Institute of Medical Sciences, Guwahati, Assam, India; ^11^Mental Health Research Center, Psychosocial Health Research Institute, Department of Psychiatry, School of Medicine, Iran University of Medical Sciences, Tehran, Iran

**Keywords:** psychiatry, art, social stigma, discrimination, mental disorders

## 1 Introduction

Stigma was first defined by Goffman in 1963 as a “spoiled identity that discredits a person in society” (1).

In the mental health context, stigma can be categorized as two-fold, internal stigma and public stigma. Public stigma occurs when members of the public endorse stereotypes about mental illness and act based on these stereotypes. It refers to a group of negative attitudes and beliefs that motivate the public to fear, reject, avoid, and discriminate against people with mental illness (2). Self-stigma is public stigma that is internalized (2). We cite these two types of stigma as a means of simplification, which is not to say that they summarize all forms of stigma, particularly as they pertain to their most-studied outcome of interest to mental health, reduction in help-seeking behavior (3).

The impact of stigma is difficult to overstate. Stigma against mental illness has been noted in multiple studies to be highly prevalent globally (4–6), and the fear of being subject to stigma has been shown to be one of the most significant barriers to accessing mental health care among a variety of populations (7–9). Meaningful methods to combat stigma and improve access to mental health resources are therefore direly needed. In the literature, different forms of art and artistic expression have been the subject of study as one possible means of combatting mental health stigma (10–15). A systematic review showed that interventions co-employing multiple art forms, including documentary, music, radio, and visual arts, are effective in combatting stigma (10). Cinema has generally been shown to be the single most effective art form in combating stigma (10, 11). Theater has also been shown to be effective (12, 13). Music has also been discussed in the literature as a tool for ameliorating stigma in public consciousness (14).

In this opinion article, we bring together perspectives from early career psychiatrists and trainees with various cultural backgrounds practicing in nine different countries, who met through their common work in a global mental health think tank. The authors were further unified by their common interests in discussing stigma against mental illness and some of the available avenues, through art, to combat that stigma on both local and national levels. The authors fall back on their own experiences in their countries of practice, and, with that, also supplement their observations, when necessary, with literature that pertains to these experiences. While the literature in this work might not be all comprehensive, it is meant to reflect on the various experiences of authors in their countries of practice.

## 2 Stigma in different countries

Authors reported that stigma against people with mental illness persists in their countries of practice, underscoring the global prevalence of this problem. The author from Italy noted that in his country, deep-seated societal misconceptions and prejudices contribute to a climate of fear, shame, and discrimination, discouraging individuals from seeking help for mental health concerns (16–18). He further stated that this stigma not only hinders early intervention and timely treatment but also perpetuates a culture of silence, making it challenging for individuals to openly discuss their mental health struggles (16–18). The author practicing in New Zealand noted that in that country, one in five individuals with mental illness have changed their behavior in some way to avoid discrimination (19). The author from Iran noted that stigma against the mentally ill originates not only from the general Iranian population (20) but also from healthcare workers (21, 22). He noted that internal medicine and cardiology trainees had shown particularly stigmatizing attitudes towards those suffering from mental illness (21).

For at least one contributor – practicing in Hungary – a paucity of research on the subject of mental health and stigma is a significant issue in Eastern European countries (23). The author did note, on the positive side, that Hungary recently initiated a national anti-stigma program. A recent research study found that medical practitioners (24) and the general public (25) in Hungary have positive and non-stigmatizing attitudes and behaviors towards patients with mental illness.

Stigma against people with mental illness was noted to be related to a reluctance to seek care in Iran, Lebanon, India, Italy, and the UK. As an example, our author from Lebanon stated that due to cultural stigma people with mental illness are often hesitant to visit mental health professionals and seek psychiatric assistance, resulting in delayed diagnosis and treatment. This reluctance to seek treatment is even more pronounced among men, who are expected to be emotionally strong, and any display of emotional distress is viewed negatively by the community. Similarly, our author from India stated that people with mental illness are reluctant to seek help due to the fear of being judged or discriminated against. People with mental illness often prefer to seek help of non-psychiatrists (e.g. general physician, neurologist) doctor, alternative medicine doctor, and faith healers due to stigma related to mental illness and by extension, psychiatrists.

Stigma may be particularly visible in certain settings. For example, authors from the UK and New Zealand noted the widespread existence of stigma in places of work and education (15, 19).

The reports by our contributors echoed trends in the global literature. Professor Sartorius, former president of the WPA, EPA and the WHO division of mental health, in an article in 2007 wrote that stigma is the central obstacle towards provision of care for people with mental illness (4). Similarly, a systematic review studying stigma in Latin America and the Caribbean found public stigma towards individuals with mental illness as well as stigma from mental health professionals in the community but less so in university settings (6).

## 3 Stigma in different languages

Many authors pointed out the pervasive use of stigmatizing language by the general public in different countries and languages, when referring to people living with mental illness, including derogatory terms like “crazy”, “nuts” or “unstable”.

In Lebanon people with mental illness may be referred to by the term “Majnoun”, a dismissive expression encapsulating any behavior outside the norms of society. In Portuguese one would state that one “has little monkeys in the attic” or “Has a screw missing” or that they “Do not play with a full deck” to refer to someone with mental illness. In Hungarian, similar words are used to describe mental illness albeit the author refers to diagnostic labels as a more common way of using stigmatized language. As an example, in Hungary, the author noted that stigma manifested itself in the frequent use of disparaging language like “schizophrenic” as opposed to “people with schizophrenia “among medical professionals, despite publication manuals of the APA and AMA recommendations suggesting the use of the latter, more humanizing term (26, 27). Our authors from Iran noted that people with mental illness are compared to the supernatural and described as devils.

On the other hand, authors from the United States of America commented on more widespread issues related to public stigma such as its relationship to race. In America, studies showed that racial and ethnic minority groups often expressed greater public and self-stigma compared to white American groups (28). They noted that similar stigma was frequently encountered in rural populations (29).

## 4 Arts to address stigma

While all authors highlighted the presence of stigma in their countries, their experience of the use of art in their local hospitals to address stigma differed substantially. Many of the authors cited art as a healing intervention for patients with mental illness, but very few noted specific efforts by their local hospital system to target mental health stigma in the local population. The exceptions were the authors from UK and New Zealand hospitals who noted local art projects such as art installations and utilizing creative arts for health promotion and to combating stigma.

Most countries exhibited a national effort to combat stigma albeit through different means. For example, the UK, India, and New Zealand cited governmental efforts to alleviate stigma through the arts. In others, there are civilian-led campaigns. In Hungary for example, the national art festival Psychart24 whose goal was to encourage people with mental illness to paint alongside members of the general population and showcase their work to promote equality. In the US, “This Is My Brave” a storytelling theatre show where individuals with mental illness highlight their stories. In the Spring of 2020, this later developed into BraveTV to bring stories of hope online during the COVID-19 pandemic. In Lebanon, there are localized efforts through various NGOs that encourage the facilitation of community mental health art projects aiming to showcase the artistic work of individuals with mental illness in the community. In Tunisia there was a famous play “Jonoun” centered on the lived experience of a patient with schizophrenia. Furthermore, in the wake of the revolution in Tunisia art has gained greater prominence within the public eye. The question of how culture influences art and its potential to mitigate stigma, both locally and nationally, prompted diverse viewpoints among the authors. This underscores the cultural diversity of the multiple nations from which our authors hail. In the USA focus was primarily on Hollywood’s cinematic depictions of mental illness. In other countries such as Italy and Hungary the relationship between architecture and culture and artworks and culture is instrumental. For example, the artworks of *Laocoön and His Sons*, the madness of Herakles and Dying Gaul were all cited. In New Zealand, Maori art forms include wood carving, weaving, tattooing, and painting. In Lebanon music, dance and literature are expressions of culture. Despite these varied perspectives, authors generally did not perceive a significant relationship between celebrating culture and mitigating the stigma against mental illness.

## 5 Conclusions

Mental illness-related stigma still exists throughout the world, but efforts are underway to combat it. These efforts take place at a variety of levels - local, civic, federal - and are as unique as the locales from which they originate. The arts have an important and growing role to play in aiding those who seek to combat the stigma against mental illness throughout the world. The authors hope that by discussing the power of art and its multiple uses throughout the world, especially with regards to its relationship to culture, that readers can be made aware of mental health stigma and the role that art can play in helping to combat it.

## Author contributions

SarE: Conceptualization, Data curation, Investigation, Methodology, Writing – original draft, Writing – review & editing. EF: Writing – original draft, Writing – review & editing. MB: Data curation, Writing – review & editing. RF: Conceptualization, Data curation, Writing – review & editing. SamE: Conceptualization, Data curation, Writing – review & editing. AL: Data curation, Writing – review & editing. DO: Data curation, Writing – review & editing. MP: Conceptualization, Data curation, Supervision, Writing – review & editing. RoR: Data curation, Writing – review & editing. RaR: Data curation, Writing – review & editing. FR: Data curation, Writing – review & editing. MS: Conceptualization, Data curation, Methodology, Supervision, Writing – review & editing.
